# A Data‐Driven Operational Definition of Square‐Wave Jerks and Its Diagnostic Utility in Progressive Supranuclear Palsy

**DOI:** 10.1111/ene.70790

**Published:** 2026-10-20

**Authors:** Duy Duan Nguyen, Andrea Quattrone, Mark Paine, Amir Fazlollahi, Soohyun Lee, Baibin (Ben) Wang, Tanya Davidson, Felicia Coleman, John D. O'Sullivan, Peter J. Nestor

**Affiliations:** ^1^ Clem Jones Centre for Ageing Dementia Research, Queensland Brain Institute The University of Queensland St Lucia Queensland Australia; ^2^ Cognitive Health Program, Mater Research Institute The University of Queensland South Brisbane Queensland Australia; ^3^ University of Medicine and Pharmacy, Hue University Hue City Vietnam; ^4^ Neuroscience Research Centre University Magna Graecia of Catanzaro Catanzaro Italy; ^5^ Department of Medsical and Surgical Sciences, Institute of Neurology University Magna Graecia of Catanzaro Catanzaro Italy; ^6^ Neuro‐Ophthalmology and Neuro‐Otology Clinic, Department of Neurology Royal Brisbane & Women's Hospital Herston Queensland Australia; ^7^ Department of Neurology Royal Brisbane & Women's Hospital Herston Queensland Australia; ^8^ Faculty of Medicine, Frazer Institute The University of Queensland Herston Queensland Australia; ^9^ Department of Neurology, Mater Adult Hospital Mater Misericordiae Limited South Brisbane Queensland Australia

**Keywords:** atypical parkinsonism, fixations, nested cross‐validation, Parkinson's disease, progressive supranuclear palsy

## Abstract

**Background:**

The Movement Disorder Society diagnostic criteria for progressive supranuclear palsy (PSP) list frequent square‐wave jerks (SWJ) as a suggestive oculomotor feature. A standardised operational definition of SWJ is however presently lacking.

**Objective:**

To create a data‐driven optimal SWJ definition and to assess its diagnostic utility in PSP.

**Methods:**

We prospectively recruited PSP *n* = 25, Parkinson's disease (PD) *n* = 32, Alzheimer's disease (AD) *n* = 35, and controls *n* = 88. SWJ were recorded with a visual fixation task using video‐oculography. Multiple candidate definitions of SWJ were generated by systematically varying morphological features, producing 32 candidate definitions. A nested cross‐validation was used to obtain unbiased diagnostic performance and optimised SWJ definition. Results were then externally tested in a fully independent sample of *n* = 25 PSP, *n* = 41 PD and *n* = 25 controls.

**Results:**

The optimal selected SWJ definition consisted of saccade amplitude 1°–8°, intra‐SWJ intervals 20–600 ms, directional opposition within ±45°, and amplitude similarity ≤ 0.25. Only SWJ rate, and not morphological features, was distinctive for PSP. SWJ rate gave out‐of‐fold AUCs of 0.81 for PSP versus controls, 0.71 for PSP versus AD, 0.63 for PSP versus PD, and 0.76 for PSP versus all non‐PSP groups. Post hoc analyses using this data‐driven definition on the external validation cohort yielded relatively comparable results. SWJ rate > 24/min had high specificity, but low sensitivity, for PSP when compared with PD.

**Conclusions:**

SWJ rate was significantly increased in PSP but with insufficient discriminant ability for use as a stand‐alone diagnostic marker. This data‐driven definition may serve as a reproducible operational definition for future research.

## Graphical abstract

A data‐driven definition of square‐wave jerks (SWJ) was developed using nested cross‐validation in 180 participants and applied unchanged to an independent cohort of 91 participants. SWJ rate was higher in progressive supranuclear palsy (PSP), whereas SWJ morphology did not distinguish diagnostic groups. Rates above 24 events/min had high specificity but low sensitivity for PSP versus Parkinson's disease, supporting use alongside other ocular motor findings rather than as a standalone diagnostic marker.

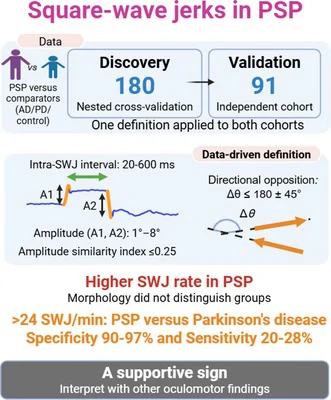

## 1 Introduction

Progressive supranuclear palsy (PSP) is a neurodegenerative disease in which oculomotor abnormalities are a core diagnostic feature. The Movement Disorder Society PSP diagnostic criteria include oculomotor dysfunction as one of the core clinical features and include within this core domain the presence of frequent macro square‐wave jerks [1]. Square‐wave jerks (SWJ) are a form of saccadic intrusion, consisting of a small saccade away from a fixation target followed, after a brief intersaccadic interval, by a corrective return saccade [2].

The operational definition of SWJ, however, is not standardised. Previous studies have used heterogeneous criteria for minimum and maximum amplitude; inter‐saccadic intervals; and constraints on directional reversal or amplitude similarity between the two saccades [2, 3, 4, 5, 6]. As these parameters determine which events are classified as SWJ, different definitions can substantially alter SWJ counts and derived morphological features. As a result, reported diagnostic performance has been inconsistent [4, 6, 7], and it remains unclear which aspects of SWJ—such as their frequency or morphological features—are most relevant for distinguishing PSP from other neurodegenerative disorders.

Despite the increasing use of automated video‐oculography in neurodegenerative research, few studies have systematically examined how alternative SWJ definitions influence the discrimination of PSP [4, 7, 8]. A further methodological concern relates to model evaluation. Multiple candidate parameterisations created by varying the SWJ definition have been explored in previous studies [4, 7]. Selecting the definition that yields the highest receiver operating characteristic area under the curve (AUC) on the same dataset may introduce selection bias, leading to inflated estimates of diagnostic performance [9, 10, 11]. Nested cross‐validation (CV) mitigates this issue by separating parameter optimisation from independent performance evaluation [12, 13].

The present study, therefore, aimed to identify a reproducible operational definition of SWJ that maximises PSP discrimination from other neurodegenerative diseases by estimating its unbiased and generalizable diagnostic performance within a nested CV framework. Then, we tested that definition on an external, fully independent cohort to assess the generalizability of the result. We further examined the underlying SWJ characteristics to determine which features drive this discrimination.

## 2 Methods

### 2.1 Study Design and Participants

This cross‐sectional study adhered to the STROBE guidelines [14]. Participants were recruited into a discovery cohort at Queensland Brain Institute, University of Queensland, Australia and an external validation cohort at the Magna Graecia University of Catanzaro, Italy.

Discovery cohort participants were prospectively recruited between September 2019 and March 2026. All patients were diagnosed by at least one of three experienced neurologists (PJN, JDO'S, MP) according to current consensus diagnostic criteria. PSP was diagnosed and subclassified based on predominant clinical features at initial presentation [1]. Eye movement recordings were obtained after probable PSP diagnostic criteria were met.

Two other neurodegenerative diseases—Parkinson's disease (PD) as a clinically relevant parkinsonian comparator and Alzheimer's disease (AD) as a non‐parkinsonian neurodegenerative comparator with cognitive impairment—were included. Probable and clinically established PD were diagnosed based on MDS criteria [15]. Clinically symptomatic AD with biomarker evidence of Alzheimer pathology—defined by either cerebrospinal fluid reduced amyloid‐β 1–42 and elevated phosphorylated tau/amyloid‐β 1–42 ratio or a positive amyloid‐PET scan—were also included [16]. Healthy controls were screened to exclude neurological or major psychiatric disease and required an Addenbrooke's Cognitive Examination III (ACE‐III) [17] score > 88/100. All participants underwent global cognitive assessment using the ACE‐III. Disease severity in PSP and PD groups was rated using the modified Hoehn and Yahr scale (H–Y) [18].

The external validation cohort comprised PSP, PD, and healthy controls aged > 50 without neurological disorders, as described in a previous publication [4]. Although participants were recruited using the same study protocol as the previous publication, the present validation cohort was not identical to the one previously reported. Global cognition was assessed using Montreal Cognitive Assessment (MoCA) corrected for age and education as previously published [19]. The validation dataset was not used during model development. The SWJ definition identified in the discovery cohort was applied unchanged to evaluate external diagnostic performance.

### 2.2 Eye‐Tracking Setup

Discovery‐cohort eye movements were recorded monocularly using an EyeLink 1000 (1000 Hz) at 100 cm with head stabilisation; calibration required mean error < 1° and maximum error < 1.5°. Validation recordings used an EyeLink Portable Duo (500 Hz) at 60 cm from a 24‐in. monitor with standard five‐point calibration. The detailed recording procedure was described in previous publications [4, 20].

### 2.3 Visual Fixation Task

In the discovery cohort, participants fixated on targets presented sequentially at five screen locations for 3.2 s each: a central target (0°, 0°) and four eccentric targets located at the corners of the display (±13.4° horizontal, ±7.7° vertical). In the validation cohort, participants performed a 5‐s central fixation task only. The fixation paradigms differed in duration and target presentation, providing an opportunity to evaluate the robustness of SWJ definitions across acquisition protocols. See Table S1 for further details.

### 2.4 Data Analysis

Eye movement traces were processed offline using a validated automated saccade detection algorithm adapted from the DEMoNS framework [5] in both discovery and validation cohorts. Blink‐contaminated segments were excluded, and saccades were identified using adaptive velocity, acceleration, and directional criteria. Effective fixation duration excluded blink‐contaminated or signal‐loss periods. Detailed parameters are provided in Protocol S1.

### 2.5 Nested CV Framework

The nested CV framework comprised six steps as described in Figure 1.

**FIGURE 1:**
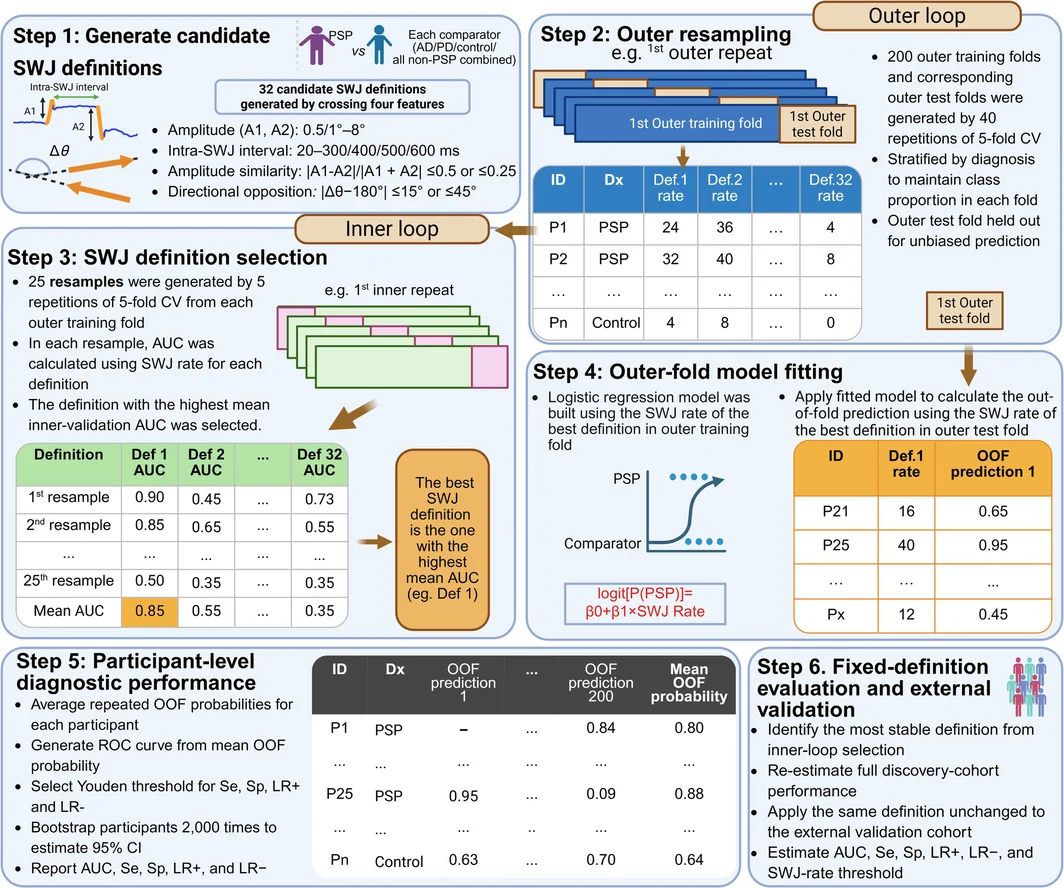
Nested CV framework for SWJ definition optimisation and performance estimation. Step 1: Generation of candidate SWJ definitions. Thirty‐two candidate SWJ definitions were generated by crossing four literature‐derived features: saccade amplitude, intra‐SWJ interval, directional opposition, and amplitude similarity (Table S2). As the putative ‘coupling’ mechanism likely happens when SWJ > 0.5° [21], the lower bound of 0.5° was chosen reducing inclusion of micro‐saccades or low‐amplitude noise, whereas the upper limit of 8° was pragmatically chosen from our experience. For each definition, SWJ rate per min—the number of SWJ × effective fixation time in seconds per minute, was calculated. Step 2: Outer resampling. For each comparison, the discovery cohort was split using 40 repetitions of 5‐fold CV, thus generating 200 outer training folds and 200 corresponding outer test folds. Splits were stratified by diagnosis to preserve the class proportion in each fold. Each outer test fold was held out and only used for unbiased assessment of predictive performance. For each outer training fold, we computed SWJ rates under all 32 candidate definitions for every participant within that fold (Example table of Def. 1 rate to Def. 32 rate). Step 3: SWJ definition selection. Within each outer training fold, we used an inner loop to select the best‐performing SWJ definition, and these were resampled using 5 repetitions of 5‐fold CV, giving 25 inner resamples. In each inner resample, AUC was calculated for each of the 32 candidate definitions, using SWJ rate as the predictor. For each outer training fold, AUCs were then averaged across the 25 inner resamples and the definition with the highest mean inner‐validation AUC selected as the ‘best’ definition for that outer fold (Example AUC table). Step 4: Outer‐fold model fitting. Using the best definition identified in the inner loop, a logistic regression model was fitted in the outer training fold, with SWJ rate as the sole predictor of PSP status. We then applied this model's coefficients to the corresponding outer test fold to obtain out‐of‐fold (OOF) predicted probabilities for each participant (Example prediction table). This procedure—inner‐loop selection, model fitting, and OOF prediction—was repeated for all 200 outer folds, so that each participant received multiple OOF predictions from different outer test fold repeats. Step 5: Participant‐level diagnostic performance. For each participant, their OOF predicted probabilities were averaged across repeats to obtain a single mean OOF probability (Example table). Using these mean OOF probabilities, we constructed ROC curves and estimated the ROC AUC. We identified the decision threshold that maximised the Youden index and used this to calculate sensitivity, specificity, positive likelihood ratio, and negative likelihood ratio. 95% confidence intervals for these measures were obtained from 2000 participant‐level bootstrap resamples with replacement. Model calibration was assessed using the Brier score, scaled Brier score, calibration intercept, calibration slope, and calibration plots. Step 6: Fixed‐definition evaluation and external validation. Finally, we identified the operational definition most frequently selected during inner‐loop optimisation across repeated resampling iterations and diagnostic comparisons. This approach prioritised stability and reproducibility over maximal performance in any single comparison, reducing the likelihood of overfitting and supporting the selection of a more generalisable SWJ definition. This fixed definition was then applied unchanged to the independent validation cohort to assess transportability and generalisability, and reapplied to the full discovery cohort to estimate clinically relevant cut‐off value of SWJ rate in the discovery cohort.

In additional analyses, we repeated with total SWJ amplitude per min (summed saccadic components × effective fixation duration in seconds per minute) used as the primary outcome instead of SWJ rate.

### 2.6 SWJ Characteristic Analysis Using the Selected Operational Definition

The definition was applied to the original dataset to extract SWJ features for each participant, including SWJ rate (events/min), mean SWJ amplitude (mean amplitude of the saccadic components, °), mean intra‐SWJ interval (ms), and mean vertical component. The vertical component is a ratio of vertical component amplitude to total amplitude of SWJ, where:

$$\text{total amplitude}=\sqrt{\text{vertical}\;{\text{component}}^{2}+\text{horizontal}\;{\text{component}}^{2}}$$

The 0‐value meaning totally horizontal and 1 meaning totally vertical, as described previously [6, 7].

### 2.7 Statistical Analysis

Continuous variables were assessed using Shapiro–Wilk tests and, because most were non‐normal, compared using non‐parametric tests. Sex distribution across groups was compared using the Chi‐square test, and H–Y stage between disease groups was compared using the Wilcoxon rank‐sum test. Differences in age, disease duration, ACE‐III or corrected MoCA scores across diagnoses were evaluated using Kruskal–Wallis tests, followed by Dunn post hoc pairwise comparisons with adjustment for multiple comparisons.

Linear regression models across diagnostic groups in both cohorts were used to compare SWJ rate, mean SWJ amplitude, mean intra‐SWJ interval, and mean vertical component across diagnoses. Healthy controls served as the reference diagnostic group in both cohorts. Unstandardised diagnostic‐group coefficients therefore represent age‐ and sex‐adjusted differences from controls in the original units of each SWJ outcome. Model assumptions were assessed; heteroscedasticity‐consistent robust standard errors were used when required. To assess sensitivity to outcome transformation, between‐group SWJ‐rate analyses were repeated using log(SWJ rate + 1), in which the addition of 1 accommodated zero rates. Original‐scale models remained the primary analyses because their coefficients represented adjusted absolute differences in mean SWJ rate.

In the discovery PSP cohort, a multivariable linear regression examined associations between SWJ rate and ACE‐III score, H–Y stage and disease duration, for which data were complete. Visual acuity was examined in a separate discovery‐cohort model because it was unavailable for 57/180 participants (*n* = 41 of whom were healthy controls). In the validation PSP cohort, incomplete clinical data prompted the use of separate univariable linear regressions examining associations between SWJ rate and PSPRS, H–Y stage, MoCA raw score, MDS‐UPDRS part III and disease duration. This approach retained the available sample for each measure, with Holm adjustment across the five tests. The mechanism of missingness could not be established.

Two‐way intraclass correlation coefficients of SWJ rate and SWJ amplitude were used to evaluate test–retest agreement for the automatic algorithm measured 3 to 12 months apart in 18 controls. Within the PD group, a linear regression model was built to investigate the effect of Levodopa equivalent daily dose on SWJ rate with H–Y score as a covariate.

All hypothesis tests were two‐sided, and *p* < 0.05 was considered statistically significant. Statistical analyses were performed using RStudio version 4.4.3.

## 3 Results

### 3.1 Demographic Information

In the discovery cohort, 25 patients with probable PSP included PSP–Richardson's syndrome (PSP–RS, *n* = 11), PSP–corticobasal syndrome (PSP–CBS, *n* = 5), PSP–frontal (PSP–F, *n* = 4), PSP–speech/language (PSP–SL, *n* = 3), PSP–progressive gait freezing (PSP–PGF, *n* = 1), and PSP–parkinsonism (PSP–P, *n* = 1). None of this PSP cohort was using Levodopa at the time of saccade recording. One PSP–CBS patient was taking Amantadine. All PD patients were using Levodopa. All PSP patients developed saccadic eye‐movement abnormalities and postural instability during longitudinal follow‐up (median follow‐up 12 [1st–3rd interquartile range: 6–22] months). The validation cohort (*n* = 25) comprised probable PSP–RS (*n* = 15), PSP–P (*n* = 6) and PSP–PGF (*n* = 4).

Across both cohorts, the PD groups showed a male predominance. Disease duration did not differ significantly between PD and PSP. H–Y did not differ between PSP and PD in the discovery cohort but was significantly higher in PSP in the validation cohort (Table 1).

**TABLE 1:** Demographics and clinical characteristics of participants.

	Discovery cohort					Validation cohort			
	Control (*n* = 88)	AD (*n* = 35)	PD (*n* = 32)	PSP (*n* = 25)	*p*	Control (*n* = 25)	PD (*n* = 41)	PSP (*n* = 25)	*p*
Age	65.5 ± 8.5	65.9 ± 7.3	70.7 ± 7.6	68.4 ± 7.1	< 0.001*	61.8 ± 6.7	62.2 ± 8.1	71.8 ± 7.6	< 0.001^#^
Sex (M|F)	36|52	20|15	25|7	11|14	0.002^§^	0|25	25|16	18|7	< 0.001^¥^
Disease duration (years)	—	3.2 ± 3.0	5.3 ± 4.8	3.9 ± 2.4	0.09	—	4.9 ± 3.2	3.3 ± 1.7	0.10
ACE‐III	94.6 ± 4.0	72.7 ± 15.7	87.6 ± 8.0	78.7 ± 13.6	< 0.001^†^	—	—	—	—
Corrected MoCA	—	—	—	—	—	20.6 ± 3.3	19.1 ± 3.2	14.7 ± 4.7	< 0.001^€^
H–Y scale	—	—	1.7 ± 1.0	2.0 ± 1.5	0.11	—	1.5 ± 0.5	3.4 ± 1.0	< 0.001^‡^

*Note:* Post hoc comparisons: *Control and AD < PD (*p* < 0.01). ^#^PSP > PD/control. ^§^Male proportion: PD > control/AD/PSP (*p* < 0.05). ^¥^Male proportion PD/PSP > control (*p* < 0.001). ^†^Control > AD, PD, PSP (*p* < 0.001) and PD > AD (*p* < 0.05). ^€^Control and PD > PSP. ^‡^PSP > PD.

### 3.2 SWJ‐Definition Stability

The definition comprising amplitudes between 1° and 8°, an intra‐SWJ interval of 20–600 ms, directional opposition within ±45°, and amplitude similarity ≤ 0.25 was the most frequently selected when comparing PSP versus AD, control, or versus all others combined (Figure 2). In contrast, definition selection was less stable for PSP versus PD where multiple combinations of directional opposition and amplitude similarity achieved comparable performance. Overall, the most frequently selected definition—directional opposition within ±45° and amplitude similarity ≤ 0.25 in addition to the above amplitude and interval ranges—was adopted for post hoc analyses and validation.

**FIGURE 2:**
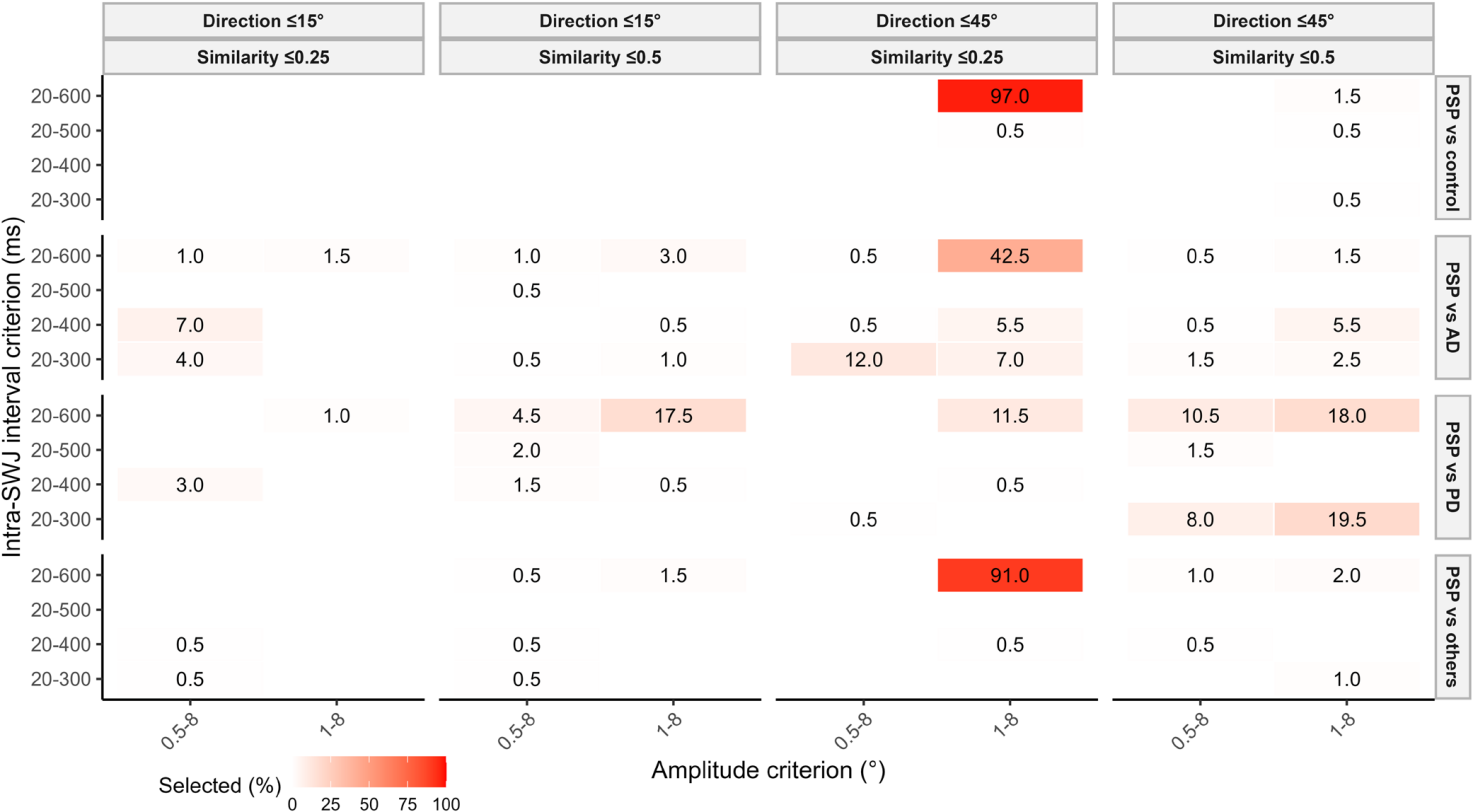
Frequency of SWJ definition being selected from inner loop across diagnostic comparisons. Number in each cell illustrating the percentage of corresponding definition being selected among 200 inner loops for each comparison.

### 3.3 SWJ Characteristics Applying the Selected Definition

Having established the operational definition of SWJ, their rate and morphological features (amplitude, intra‐SWJ interval and vertical component) were contrasted across groups (Figure 3). Linear regression models adjusted for centred age and sex showed that age and sex were not significantly associated with any SWJ characteristics. SWJ rate in PSP (17.7 ± 17.6) was significantly higher than in controls (3.7 ± 6.6), PD (7.2 ± 6.9) and AD (6.4 ± 10.1). There were no significant differences in mean SWJ amplitude, mean intra‐SWJ interval and mean vertical component (Figure 3A, Table S3A–E). SWJ rate did not differ significantly across the five fixation positions across all groups in the discovery cohort (Figure S1, Table S3F).

**FIGURE 3:**
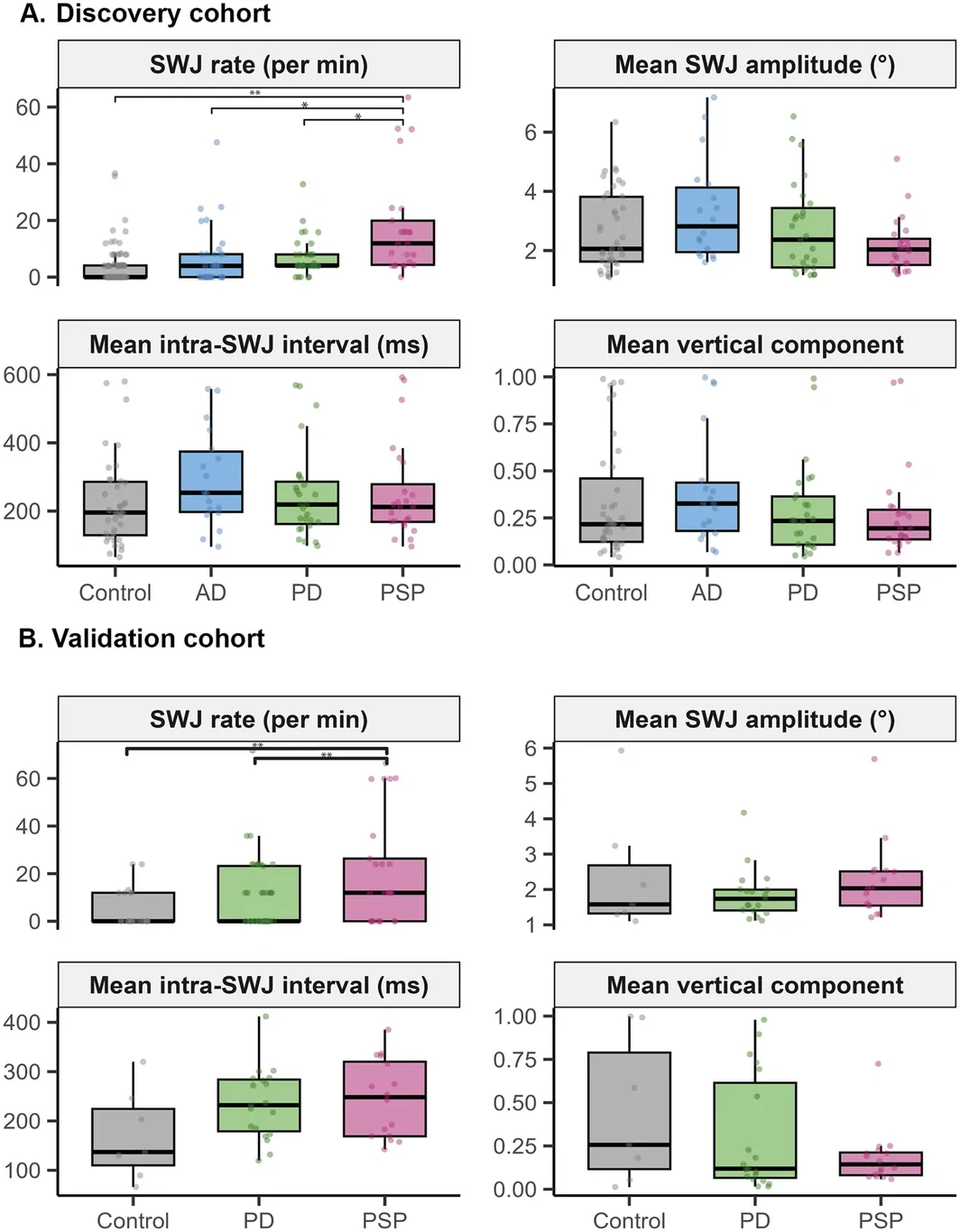
SWJ characteristics in discovery cohort (A) and validation cohort (B). *p < 0.05. **p < 0.001.

Validation findings were similar: PSP had higher SWJ rates than PD and controls, without differences in amplitude, intra‐SWJ interval, or vertical component (Figure 3B, Table S4A–E).

### 3.4 Covariate, Test–Retest Reliability and Phenotypes Analyses

Visual acuity was not significantly associated with SWJ rate across groups (Table S5A). In the PD group, there was no significant relationship between SWJ rate and Levodopa equivalent daily dose (Table S5B). Within the PSP group in the discovery cohort, SWJ rate was not significantly associated with ACE‐III score, disease duration or H–Y score (Table S5C). Likewise, none of the clinical severity scales were significant predictors of SWJ rate in PSP in the validation cohort (Table S5D). The test–retest reliability of SWJ rate in controls was found to be excellent (ICC = 0.95, *p* < 0.001) (Table S5E).

A subgroup analysis comparing PSP–RS versus PSP non‐Richardson's phenotypes found no significant difference in SWJ rate in the discovery cohort, whereas higher SWJ rates were found in PSP‐RS in the validation cohort (Table S5F, Figure S2).

### 3.5 Diagnostic Performance and External Validation of SWJ Rate

In the nested CV model, the participant‐level diagnostic performance of SWJ rate showed good discrimination between PSP and controls or AD, but performed less well at distinguishing PSP from PD. When all non‐PSP participants were combined into a single comparison group, overall discrimination remained good. The likelihood ratios of SWJ rate provided moderate diagnostic evidence (Table 2A).

**TABLE 2:** Internal and external diagnostic performance of SWJ rate for discriminating PSP from comparator groups.

(A) Discovery cohort: nested‐CV participant‐level OOF estimate					
PSP versus	AUC (95% CI)	Sensitivity (95% CI)	Specificity (95% CI)	LR+ (95% CI)	LR− (95% CI)
Control (*n* = 88)	0.81 (0.70–0.90)	0.84 (0.56–1.00)	0.78 (0.50–0.94)	3.52 (1.89–12.32)	0.21 (0–0.47)
PD (*n* = 32)	0.63 (0.50–0.77)	0.60 (0.20–0.92)	0.75 (0.31–1.00)	2.35 (1.30–10.20)	0.55 (0.20–0.82)
AD (*n* = 35)	0.71 (0.58–0.83)	0.96 (0.52–1.00)	0.54 (0.37–0.91)	2.10 (1.52–6.53)	0.09 (0.00–0.56)
Others (*n* = 155)	0.76 (0.65–0.85)	0.76 (0.52–1.00)	0.74 (0.41–0.91)	2.96 (1.62–6.96)	0.32 (0–0.56)

(B) Selected definition, full‐cohort post hoc analysis						
Cohort	PSP versus	AUC	Sensitivity	Specificity	LR+	LR−
Discovery	PD (*n* = 32)	0.72	0.60	0.78	2.74	0.51
	Control (*n* = 88)	0.85	0.84	0.75	3.36	0.21
Validation	PD (*n* = 41)	0.63	0.60	0.63	1.64	0.63
	Control (*n* = 25)	0.69	0.60	0.76	2.50	0.53

Abbreviations: AUC, area under the curve of receiver operating characteristic; CI, confidence interval; LR−, negative likelihood ratio; LR+, positive likelihood ratio; OOF, out‐of‐fold.

Model calibration was generally acceptable across diagnostic comparisons. Brier scores were lower for PSP versus controls and PSP versus all non‐PSP groups, whereas higher Brier scores were observed for PSP versus PD and PSP versus AD. Calibration plots showed broadly acceptable agreement between predicted and observed PSP probabilities (Table S6, Figure S3).

When applying the selected SWJ definition to the validation cohort, AUC for PSP versus PD remained similar whereas AUC for PSP versus control reduced to the lower bound of the 95% confidence interval of the corresponding nested CV AUC. The performances for PSP versus controls were higher than for PSP versus PD in both cohorts (Table 2B).

When comparing PSP versus PD, at the threshold of > 24 SWJ per min the sensitivity was 20% and specificity was 97% in the discovery cohort. Similar results were found in the validation cohort (28% sensitivity, 90% specificity). Applying a threshold of > 10 SWJ per min (as mentioned in the MDS diagnostic criteria for PSP) sensitivities were 56% in discovery and 60% in validation cohorts with specificities of 78% in discovery and 54% in validation (Figure S4).

Additional models: using total SWJ amplitude per min as the outcome within the same nested CV framework did not yield superior performance compared with SWJ rate (Table S7).

## 4 Discussion

Across repeated optimisation procedures, a definition requiring paired saccades of 1°–8° in amplitude, an inter‐saccadic interval of 20–600 ms, directional opposition within ±45°, and amplitude similarity ≤ 0.25 emerged as the most consistently selected criterion set. Importantly, post hoc analyses demonstrated that the principal distinguishing feature of PSP was increased SWJ frequency rather than altered SWJ morphology. SWJ provided meaningful but only modest diagnostic information: highest when distinguishing PSP from controls and AD, whereas discrimination from PD was considerably weaker with similar results identified in the validation cohort.

### 4.1 Operational Definition of SWJ

The nested CV procedure selected the SWJ definition according to diagnostic discrimination rather than event yield. More inclusive definitions, such as a lower amplitude threshold of 0.5° or less stringent amplitude‐similarity constraints, were not favoured because they increased SWJ counts across diagnostic groups without improving discrimination. For PSP versus controls and PSP versus all non‐PSP groups, only a small number of alternative definitions were repeatedly selected, supporting the stability of the discriminative signal. In contrast, criterion selection was more variable for PSP versus PD, consistent with the weaker calibration and lower diagnostic performance in this comparison. Therefore, the final operational definition was guided primarily by the more stable comparisons, while recognising that SWJ rate has more limited utility for distinguishing PSP from PD. The 1°–8° amplitude range also aligned with the clinical concept of the macro‐SWJ, which is visible during bedside examination while saccade amplitudes < 0.5° are too small to be visible on clinical bedside testing [22].

### 4.2 SWJ Characteristics Using the Selected Operational Definition

The SWJ frequency, but not SWJ morphology (i.e., amplitude, intra‐SWJ interval, or vertical component), was significantly different in PSP compared to other groups providing insight into the underlying pathophysiology of fixation instability. A plausible interpretation is that within PSP, degeneration affecting networks involved in fixation control—potentially including superior colliculus, mesencephalic reticular structures, cerebellar pathways, and their cortical inputs—may reduce fixation stability and increase the occurrence of intrusive saccades [3, 6, 23, 24, 25, 26]. Nonetheless, once an intrusive saccade has occurred, the corrective process that generates the return component of the SWJ (i.e., so called ‘coupling’ mechanism) [21] appeared similar across all groups. The findings were consistent with previous studies that found the intra‐SWJ interval in PSP to be similar to that of controls [7], PD, multiple system atrophy, corticobasal degeneration, and spinocerebellar ataxia [27]. At odds with the current findings, however, one previous study reported that SWJ in PSP were larger and more horizontally biased than in controls [7]. These discrepancies with our findings likely reflect differences in the SWJ definition. Otero‐Millan et al. built an automatic approach, in which directional opposition, intra‐SWJ interval and amplitude similarity were weighted equally to derive the SWJ likelihood [7]. Although the model captured the continuum of saccadic intrusions in PSP, it could be hard to interpret clinically. Our SWJ approach can be interpreted as a diagnostic operational definition of clinically conspicuous paired SWJs rather than a universal physiological boundary between SWJs and other fixational saccades.

### 4.3 Diagnostic Performance

Regarding diagnostic performance, discrimination between PSP and PD was weaker than discrimination between PSP and AD or controls. The inclusion of AD demonstrated that the increased SWJ rate in PSP was not simply attributable to cognitive impairment. The weaker discrimination from PD indicated that increased SWJ frequency was not specific to PSP, which is biologically and clinically plausible. Previous studies have reported increased saccadic intrusions across several parkinsonian and related movement disorders, including PD, multiple system atrophy, cerebellar disorders, and other atypical parkinsonian syndromes [3, 4, 6, 7, 8, 27, 28]. Therefore, although SWJ rate was significantly increased in PSP, overlap with PD reduced the diagnostic utility in this contrast.

When the final SWJ definition was applied to the discovery cohort and then unchanged to the independent validation cohort, the observed diagnostic performance remained broadly within the range predicted from the nested CV estimates. This supports the generalizability of the selected SWJ definition. The marginally lower performance in the validation cohort is unsurprising, given that the SWJ definition was derived from the discovery cohort and therefore remains partly optimised to that dataset. Importantly, model calibration, however, showed acceptable agreement between predicted and observed diagnostic probabilities, suggesting that this modest reduction in discrimination was not accompanied by systematic over‐ or underestimation.

Facchin et al. reported similar AUC for the number of SWJs, a raw count analogous to SWJ rate but not normalised by effective fixation duration. In their study, SWJs were identified by an expert panel, with AUCs of 0.73–0.80 for PSP versus controls and 0.66–0.76 for PSP versus PD [4]. These values were within the 95% confidence intervals from our nested CV framework and were comparable to the performance observed in the validation cohort, which used a similar experimental paradigm. This concordance suggests that increased SWJ frequency is a relatively robust and consistent finding across expert visual identification and automated detection approaches.

In contrast, Facchin et al. reported SWJ amplitude as the strongest discriminative parameter whereas it did not outperform SWJ rate in the present study. This discrepancy may relate to methodological differences in amplitude quantification. In their study, SWJ amplitude was manually quantified after expert visual identification of events, whereas our study used a fully automated event‐detection algorithm with a pre‐specified definition. Prior methodological work in eye tracking has shown that both algorithm choice and human classification rules can materially affect event boundaries and derived parameters such as duration and amplitude [29]. Therefore, SWJ amplitude may be more method‐dependent than SWJ rate. Our findings do not necessarily refute the presence of larger visually apparent SWJs in PSP; rather, they suggest that when SWJ are defined by a reproducible definition and evaluated with cross‐validation, increased event frequency is the more robust diagnostic signal. The SWJ rate did not relate to clinical severity scales in PSP and was not associated with Levodopa dose in PD, which are aligned with previous studies [4, 25].

### 4.4 Clinical Implications

From a clinical perspective, the likelihood ratios observed in this study indicated that SWJ rate should not be used as a stand‐alone diagnostic marker. SWJ rate was, however, significantly increased in PSP, suggesting it could have a diagnostic role when considered in combination with other ocular motor abnormalities, particularly vertical saccadic slowing and gaze palsy [1, 22]. Although the MDS diagnostic criteria did not set precise cut‐offs for the use of SWJ in diagnosing PSP, a threshold of > 10 SWJs/min was mentioned as being more indicative of PSP [1]; this threshold, however, showed limited specificity in our data. In contrast, higher SWJ rates of approximately > 24 events/min were highly specific (> 90%) for PSP versus PD in both cohorts, although sensitivity was low. Thus, markedly elevated SWJ frequency may be useful as a rule‐in sign when present, but a lower SWJ rate does not exclude PSP. The MDS criteria also suggested that SWJs were of higher amplitude in PSP (up to 3° to 4°), though support for this criterion was not found in the present study. Indeed, at the bedside, amplitude is more difficult to estimate without the use of video‐oculography; as such, the elevated SWJ frequency may be the only useful sign in routine clinical practice. In terms of implementing the present findings to bedside examination, the threshold of > 24/min means that the examiner needs to be seeing virtually continuous SWJ (occurring every couple of seconds) to raise concern for PSP.

### 4.5 Strengths and Limitations

Strengths include multiple PSP variants, use of nested CV to limit optimistic bias, external validation under a different acquisition protocol, and objective automated SWJ measurement. Pooling PSP subtypes was intentional and aligned with the primary aim of the study, which was to characterise SWJ across the spectrum of those meeting criteria for probable PSP, rather than to define subtype‐specific signatures.

The study has limitations: (i) diagnoses of PSP and PD were based on clinical criteria and were not pathologically confirmed; (ii) testing the patient without eyewear might reduce the effective correction although we showed that there was no effect of visual acuity on the SWJ rate; (iii) although an external cohort was included, the validation cohort used a different fixation paradigm and recording configuration. Nonetheless, in the discovery cohort, SWJ rate did not differ significantly across the five fixation positions, suggesting that the signal was not driven by target location. This supports the robustness of the finding across fixation conditions, although differences in acquisition protocol may still have contributed to the lower diagnostic performance in the validation cohort.

The definition of SWJ established in this study can now be applied to prospective multicentre studies in larger cohorts, including early disease, different PSP phenotypes and other atypical parkinsonian disorders. Also, studies should determine whether SWJ rate adds diagnostic information beyond established ocular motor signs, particularly saccadic slowing and vertical gaze limitation.

## 5 Conclusion

Applying a robust and generalisable analytical framework using nested CV enabled definition of the most informative SWJ criteria whilst minimising overfitting. Increased SWJ frequency, but not morphology, was indicative of PSP although this increased rate of SWJs was of insufficient discriminant value to be useful as a stand‐alone diagnostic marker for PSP. Though lacking sensitivity, very high SWJ rates (> 24/min) however appeared to offer high specificity for a diagnosis of PSP.

## Author Contributions

**Duy Duan Nguyen:** conceptualization, investigation, data curation, methodology, validation, visualization, writing – original draft, software. **Andrea Quattrone:** resources, validation, data curation, writing – review and editing. **Mark Paine:** conceptualization, methodology, resources, writing – review and editing. **Amir Fazlollahi:** methodology, supervision, writing – review and editing. **Soohyun Lee:** resources, data curation, funding acquisition, project administration. **Baibin (Ben) Wang:** formal analysis, methodology, writing – review and editing. **Tanya Davidson:** writing – review and editing, visualization. **Felicia Coleman:** resources, data curation, writing – review and editing. **John D. O'Sullivan:** writing – review and editing, supervision, conceptualization. **Peter J. Nestor:** conceptualization, writing – original draft, funding acquisition, project administration, supervision, methodology.

## Funding

This study was supported by NHMRC Ideas grant 2023/GNT2030157 and Mater Foundation.

## Ethics Statement

The study was approved by the Mater Misericordiae Ltd. Human Ethics Committee (EC00332) and the Magna Graecia University Ethics Committee.

## Consent

The procedures used in this study adhere to the tenets of the Declaration of Helsinki. All participants gave informed consent prior to participation.

## Conflicts of Interest

J.D.O. has received fellowship funding from Ipsen to his institution; advisory board honoraria from STADA and AbbVie; lecture honoraria from AbbVie, Ipsen, and STADA; and travel support and speaker fees from the Movement Disorders Society. P.J.N. receives research funds from NHMRC and Mater Foundation. The other authors declare no conflicts of interest.

## Supporting information


**Figure S1:** Log(SWJ rate per min + 1) across fixation positions across diagnoses.
**Figure S2:** SWJ rate per min distribution for each PSP phenotype.
**Figure S3:** Calibration plot across pairs of comparison.
**Figure S4:** ROC curves using selected definition to differentiate PSP versus PD from the discovery cohort (on the left panel) and the validation cohort (on the right panel).
**Table S1:** Comparison of eye‐tracking setup and fixation paradigms between discovery and external validation cohorts.
**Table S2:** Candidate thresholds for different SWJ features based on previous literature.
**Table S3A:** The linear model comparing SWJ rate across diagnoses including age and sex as covariates in discovery cohort.
**Table S3B:** The sensitivity analysis using linear model comparing log(SWJ rate + 1) across diagnoses including age and sex as covariates in discovery cohort.
**Table S3C:** The linear model comparing mean SWJ amplitude across diagnoses including age and sex as covariates in discovery cohort.
**Table S3D:** Linear model comparing intra‐SWJ interval across diagnoses including age and sex as covariates.
**Table S3E:** The linear model comparing mean vertical component across diagnoses including age and sex as covariates.
**Table S3F:** Linear mixed effect model comparing log(SWJ rate+1) across different fixation positions with diagnostic interactions and random intercept of each participant.
**Table S4A:** The linear model comparing SWJ rate across diagnoses including age and sex as covariates in the validation cohort.
**Table S4B:** The sensitivity analysis using linear model comparing log(SWJ rate + 1) across diagnoses including age and sex as covariates in discovery cohort.
**Table S4C:** The linear model comparing mean SWJ amplitude across diagnoses including age and sex as covariates in the validation cohort.
**Table S4D:** The linear model comparing intra‐SWJ interval across diagnoses including age and sex as covariates in the validation cohort.
**Table S4E:** The linear model comparing mean vertical component across diagnoses including age and sex as covariates in the validation cohort.
**Table S5A:** The linear model exploring the effect of visual acuity on SWJ rate across diagnoses in the discovery cohort.
**Table S5B:** The linear model exploring the effect of Levodopa dosage on SWJ rate in Parkinson's disease group in the discovery cohort.
**Table S5C:** Linear model exploring the effect of ACE‐III, Hoen‐Yahr stage, and disease duration on SWJ rate in PSP in the discovery cohort.
**Table S5D:** Exploratory associations between clinical measures and SWJ rate in the validation PSP cohort.
**Table S5E:** Test–retest reliability in the 18 controls with repeat from 3 months to 1 year in the discovery cohort.
**Table S5F:** Exploratory comparison of SWJ rate between PSP‐RS and pooled non‐RS phenotypes within each cohort.
**Table S6:** Calibration results of the nested CV model in the discovery cohort.
**Table S7:** Participant‐level diagnostic performance of SWJ in differentiating PSP from other groups using total SWJ amplitude per min in the discovery cohort.
**Protocol S1:** SWJ detection algorithm.

## Data Availability

The data that support the findings of this study are available on request from the corresponding author. The data are not publicly available due to privacy or ethical restrictions. The code for data analyses is shared in this repository: https://github.com/DuyDuanNguyen/SWJ_Operational_definition.git.
