# Supplementary material for: A Data‐Driven Operational Definition of Square‐Wave Jerks and Its Diagnostic Utility in Progressive Supranuclear Palsy
Source: Eur J Neurol. 2026 Oct 20;33(10):e70790. doi: 10.1111/ene.70790 (PMC13681254; doi:10.1111/ene.70790)
Supplement: Supplementary file 1 [file ENE-33-e70790-s001.docx]

# **Supplementary**

## **Table S1.** Comparison of eye-tracking setup and fixation paradigms between discovery and external validation cohorts

| **Domain** | **Discovery cohort** | **Validation cohort** |
| --- | --- | --- |
| Site | Queensland Brain Institute / Mater cohort | Neuroscience Research Centre, University of Catanzaro, Italy |
| Role in study | Model development, nested CV, SWJ definition optimisation | Independent external validation |
| Diagnostic groups | PSP, PD, AD, healthy controls | PSP, PD, healthy controls |
| Eye tracker | EyeLink 1000  Screen: 2560 × 1440 resolution | EyeLink Portable Duo  Screen: 1024 × 768-pixel monitor (41.6 × 23.5 cm) |
| Sampling rate | 1000 Hz | 500 Hz |
| Recording mode | Monocular | Monocular |
| Viewing distance | 100 cm | 60 cm |
| Calibration | 9-point calibration, accepted validation: mean fixation error <1.0°; maximum error <1.5° | Only 5-point calibration |
| Fixation task | Centre and four eccentric corner targets sequentially ±13.4° horizontal, ±7.7° vertical | Single central fixation Central target only |
| Duration of task | 3.2 s per target, 16s in total | 5s in total |
| Task instruction | Maintain fixation until target moved, then shift gaze to new target | Maintain fixation on central target |
| Analysis role | SWJ definition generated and optimised using nested CV | Final selected SWJ definition applied unchanged |

## **Protocol S1.** SWJ detection algorithm

Eye movement preprocessing and SWJ detection, adapted from DeMONS protocol ^5^

***Step 1. Signal preprocessing and artefact removal***

Raw gaze position traces were low pass filtered prior to event detection. Periods affected by blinks, track loss, or signal interruption were identified automatically and excluded from subsequent analyses together with a short temporal buffer surrounding each artefact.

***Step 2. Saccade detection***

Candidate saccadic intervals were initially identified using an adaptive acceleration threshold exceeding 2.576 standard deviations of the acceleration distribution. Adjacent intervals separated by less than 20 ms were merged. Saccade onset and offset were subsequently refined using velocity and directional definition, including a minimum velocity threshold of 5°/s, directional deviations exceeding 20° from the local trajectory, and directional changes exceeding 60° from the principal movement direction. Only events with a duration of at least 8 ms and an amplitude greater than 0.5° were retained as valid saccades.

## **Table S2.** Candidate thresholds for different SWJ features of based on previous literature ^2, 4, 5, 7, 8, 24^

| **SWJ features** | **Definition** | **Ranges evaluated** |
| --- | --- | --- |
| Amplitude (°) | Minimum and maximum amplitude required for both saccades in the pair. ^4, 6, 7, 20^ | 0.5-8°, 1-8° |
| Intra-SWJ interval (ms) | Time window between the end of the first saccade and onset of the return saccade. ^4, 7^ | 20-300, 20-400, 20–500, 20–600 |
| Amplitude similarity limit | Ensures similar amplitude between the two saccades in a pair (A1: amplitude of first saccade, A2: amplitude of second saccade). ^7^ | \|A1-A2\|/\|A1 + A2\| ≤0.5 or ≤0.25 |
| Directional opposition \|Δθ - 180º\| | Degree to which the two saccades must be in opposite directions (θ1: direction of 1^st^ saccade, θ2: direction of 2^nd^ saccade) ^7^ | Δθ = \|θ1 - θ2\|  \|Δθ - 180º\| ≤ 15º, ≤ 45º |

**Post hoc-SWJ characteristics analyses for discovery cohort**

## **Table S3A.** The linear model comparing SWJ rate across diagnoses including age and sex as covariates in discovery cohort

| **Part 1. Primary model**  **Model:** SWJ rate ~ Centralized age + Sex + Diagnosis  **Reference group:** Mean age of 66.89 \| Male \| Control | | | | |
| --- | --- | --- | --- | --- |
| **Variables** | **B** | **SE** | **t-value** | **p** |
| Intercept | 4.68 | 1.40 | 3.33 | 0.001 |
| Centralized age | 0.03 | 0.08 | 0.32 | 0.75 |
| Female | -1.54 | 1.68 | -0.92 | 0.36 |
| *AD* | 2.37 | 1.86 | 1.27 | 0.20 |
| *PD* | 2.71 | 1.80 | 1.50 | 0.13 |
| *PSP* | 13.82 | 3.74 | 3.70 | <0.001 |
| **Part 2. Post-hoc pairwise comparisons using Holm method** | | | | |
| **Pairwise comparison** | **B** | **SE** | **t-value** | **p** |
| Control – AD | -2.37 | 1.86 | -1.27 | 0.41 |
| Control – PD | -2.71 | 1.80 | -1.50 | 0.40 |
| Control – PSP | -13.82 | 3.74 | -3.70 | 0.002 |
| AD – PD | -0.34 | 2.35 | -0.15 | 0.88 |
| AD – PSP | -11.45 | 4.08 | -2.80 | 0.03 |
| PD – PSP | -11.11 | 3.97 | -2.80 | 0.03 |

*Note:* B: unstandardized coefficients; SE: standard error

*This model showed the heteroskedasticity, so the cluster robust type-3 was used to estimate the standard error and p-value.

*** Comments:** Age/sex was not associated with SWJ rate. PSP had 13.8 SWJ higher than control while as AD and PD showed numerically more SWJ than control but non-significant (p = 0.20 and p = 0.13, respectively) in Part 1. PSP had 11.45 and 11.11 SWJ significantly higher than AD and PD respectively (Part 2).

**Table S3B.The sensitivity analysis using linear model comparing log(SWJ rate + 1) across diagnoses including age and sex as covariates in discovery cohort**

Model: **log(SWJ rate+1) ~ Centralized age + Sex + Diagnosis**

Reference group: **Mean age of 66.89 | Male | Control**

| **Variables** | **B** | **SE** | **t-value** | **p** |
| --- | --- | --- | --- | --- |
| Intercept | 0.971 | 0.167 | 5.820 | <0.001 |
| Centralized age | 0.001 | 0.012 | 0.083 | 0.934 |
| Female | -0.110 | 0.180 | -0.611 | 0.542 |
| *AD* | 0.279 | 0.251 | 1.110 | 0.268 |
| *PD* | 0.800 | 0.229 | 3.491 | <0.001 |
| *PSP* | 1.608 | 0.243 | 6.604 | <0.00001 |

*Note:* B: unstandardized coefficients; SE: standard error

**** Comments:** Table S3B sensitivity analysis confirmed similar results to the main linear regression model at Table S3A, that was PSP had higher SWJ rate than comparators (PD, Control and AD)

## **Table S3C.** The linear model comparing mean SWJ amplitude across diagnoses including age and sex as covariates in discovery cohort

**Model:** Mean SWJ amplitude~ Centralized age + Sex + Diagnosis

**Reference group:** Mean age of 66.89 | Male | Control

| **Variables** | **B** | **SE** | **t-value** | **p** |
| --- | --- | --- | --- | --- |
| Intercept | 2.43 | 0.28 | 8.63 | <0.0001 |
| Centralized age | 0.001 | 0.02 | 0.07 | 0.94 |
| Female | 0.42 | 0.29 | 1.47 | 0.14 |
| *AD* | 0.76 | 0.40 | 1.91 | 0.06 |
| *PD* | 0.20 | 0.38 | 0.52 | 0.60 |
| *PSP* | -0.53 | 0.37 | -1.45 | 0.15 |

*Note:* B: unstandardized coefficients; SE: standard error.

**** Comments:** Mean SWJ amplitude was not significantly different across groups. PSP showed the trend of having smaller mean SWJ amplitude compared to control (B= -0.53, p=0.15)

## **Table S3D.** Linear model comparing intra-SWJ interval across diagnoses including age and sex as covariates

**Model:** Mean intra-SWJ interval ~ Centralized age + Sex + Diagnosis

**Reference group:** Mean age of 66.89 | Male | Control.

| **Variables** | **B** | **SE** | **t-value** | **p** |
| --- | --- | --- | --- | --- |
| Intercept | 204.74 | 27.58 | 7.42 | <0.0001 |
| Centralized age | -1.92 | 2.07 | -0.93 | 0.36 |
| Female | 30.68 | 28.05 | 1.09 | 0.28 |
| *AD* | 72.92 | 39.23 | 1.86 | 0.07 |
| *PD* | 44.21 | 37.25 | 1.19 | 0.24 |
| *PSP* | 34.70 | 35.85 | 0.97 | 0.34 |

*Note:* B: unstandardized coefficients; SE: standard error

**** Interpretation:** AD showed a trend toward higher intra-SWJ interval compared to control (p=0.07).

## **Table S3E.** The linear model comparing mean vertical component across diagnoses including age and sex as covariates

**Model:** Mean vertical component ~ Centralized age + Sex + Diagnosis

**Reference group:** Mean age of 66.89 | Male | Control

| **Variables** | **B** | **SE** | **t-value** | **p** |
| --- | --- | --- | --- | --- |
| Intercept | 0.27 | 0.06 | 4.94 | <0.001 |
| Centralized age | 0.003 | 0.004 | 0.89 | 0.38 |
| Female | 0.11 | 0.06 | 1.99 | 0.05 |
| *AD* | 0.11 | 0.08 | 1.33 | 1.88 |
| *PD* | -0.03 | 0.08 | -0.39 | 0.69 |
| *PSP* | -0.08 | 0.07 | -1.06 | 0.29 |

*Note:* B: unstandardized coefficients; SE: standard error

**** Interpretation:** mean vertical component was not separated between groups suggesting that SWJ in PSP was not horizontally dominated and not a distinctive feature for PSP.

## **Figure S1.** Log(SWJ rate per min + 1) across fixation positions across diagnoses.

*** The log(SWJ rate + 1) was used to adjust for the skewness of the raw data SWJ rate per min.*

## **Table S3F.** Linear mixed effect model comparing log(SWJ rate+1) across different fixation positions with diagnostic interactions and random intercept of each participant.

Model: Log(SWJ rate+1) ~ Fixation position * Diagnosis + (1|ID)

Reference group: Control | Centre fixation

| **Variables** | **B** | **SE** | **t-value** | **p** |
| --- | --- | --- | --- | --- |
| Intercept | 0.58 | 0.14 | 4.07 | <0.001 |
| **Fixation position** | | | | |
| *Top-left* | -0.02 | 0.17 | -0.11 | 0.92 |
| *Top-right* | -0.17 | 0.17 | -1.01 | 0.31 |
| *Bottom-right* | -0.25 | 0.17 | -1.47 | 0.14 |
| *Bottom-left* | 0.04 | 0.17 | 0.24 | 0.81 |
| **Diagnosis** | | | | |
| *AD* | 0.18 | 0.27 | 0.67 | 0.50 |
| *PD* | 0.31 | 0.27 | 1.12 | 0.26 |
| *PSP* | 1.04 | 0.30 | 3.46 | <0.001 |
| **Fixation position x Diagnosis** | | | | |
| *Top-left * AD* | -0.08 | 0.32 | -0.25 | 0.80 |
| *Top-right * AD* | -0.01 | 0.32 | -0.02 | 0.98 |
| *Bottom-right * AD* | -0.12 | 0.32 | -0.39 | 0.70 |
| *Bottom-left * AD* | 0.23 | 0.32 | 0.72 | 0.48 |
| *Top-left * PD* | 0.17 | 0.33 | 0.53 | 0.60 |
| *Top-right * PD* | -0.03 | 0.33 | -0.08 | 0.94 |
| *Bottom-right * PD* | -0.04 | 0.33 | -0.13 | 0.90 |
| *Bottom-left * PD* | 0.62 | 0.33 | 1.87 | 0.06 |
| *Top-left * PSP* | 0.31 | 0.37 | 0.84 | 0.40 |
| *Top-right * PSP* | 0.03 | 0.37 | 0.07 | 0.94 |
| *Bottom-right * PSP* | 0.62 | 0.37 | 1.69 | 0.09 |
| *Bottom-left * PSP* | -0.15 | 0.37 | -0.41 | 0.68 |

**** Interpretation:**

The model showed that SWJ rate per min was not dependent on the fixation positions and that was true for all diagnoses.

**Post hoc-SWJ characteristics analyses for the validation cohort**

## **Table S4A.** The linear model comparing SWJ rate across diagnoses including age and sex as covariates in the validation cohort

| **Part 1. Primary model**  **Model:** SWJ rate ~ Centralized age + Sex + Diagnosis  **Reference group:** Mean age of 64.62 \| Male \| Control | | | | |
| --- | --- | --- | --- | --- |
| **Variables** | **B** | **SE** | **t-value** | **p** |
| Intercept | 1.99 | 5.20 | 0.38 | 0.70 |
| Centralized age | -0.09 | 0.24 | -0.40 | 0.69 |
| Female | 3.51 | 4.77 | 0.74 | 0.45 |
| *PD* | 6.88 | 3.81 | 1.81 | 0.07 |
| *PSP* | 18.14 | 6.96 | 2.61 | 0.01 |
| **Part 2. Post-hoc pairwise comparisons using Holm method** | | | | |
| **Pairwise comparison** | **B** | **SE** | **t-value** | **p** |
| Control – PD | -6.88 | 1.86 | -3.69 | <0.001 |
| Control – PSP | -18.14 | 1.80 | -10.06 | <0.001 |
| PD – PSP | -11.26 | 2.35 | -4.80 | <0.001 |

*Note:* B: unstandardized coefficients; SE: standard error

*This model showed the heteroskedasticity, so the cluster robust type-3 was used to estimate the standard error and p-value.

**** Comments:** Age/sex was not associated with SWJ rate. PSP had 18.14 SWJ higher than control while as PD showed a trend of having more SWJ (p =0.07, in Part 1). PSP had 11.26 SWJ significantly higher than PD (Part 2).

## **Table S4B. The sensitivity analysis using linear model comparing log(SWJ rate + 1) across diagnoses including age and sex as covariates in discovery cohort**

Model: **log(SWJ rate+1) ~ Centralized age + Sex + Diagnosis**

Reference group: **Mean age of 64.62 | Male | Control**

| **Variables** | **B** | **SE** | **t-value** | **p** |
| --- | --- | --- | --- | --- |
| Intercept | 0.580 | 0.527 | 1.101 | 0.274 |
| Centralized age | 0.004 | 0.023 | 0.183 | 0.855 |
| Female | 0.437 | 0.425 | 1.028 | 0.307 |
| *PD* | 0.667 | 0.457 | 1.460 | 0.148 |
| *PSP* | 1.371 | 0.600 | 2.283 | 0.025 |

*Note:* B: unstandardized coefficients; SE: standard error

**** Comments:** Table S4B sensitivity analysis confirmed similar results to the main linear regression model at Table S4A, that was PSP had higher SWJ rate than comparators (PD, Control and AD)

**Table S4C.** The linear model comparing mean SWJ amplitude across diagnoses including age and sex as covariates in the validation cohort

**Model:** Mean SWJ amplitude~ Centralized age + Sex + Diagnosis

**Reference group:** Mean age of 64.62 | Male | Control

| **Variables** | **B** | **SE** | **t-value** | **p** |
| --- | --- | --- | --- | --- |
| Intercept | 1.59 | 0.51 | 3.12 | 0.003 |
| Centralized age | 0.001 | 0.02 | 0.035 | 0.97 |
| Female | 0.58 | 0.37 | 1.59 | 0.12 |
| *PD* | -0.03 | 0.48 | 0.06 | 0.95 |
| *PSP* | 0.44 | 0.50 | 0.87 | 0.39 |

*Note:* B: unstandardized coefficients; SE: standard error

** Comments: Mean SWJ amplitude was not significantly different across groups.

## **Table S4D.** The linear model comparing intra-SWJ interval across diagnoses including age and sex as covariates in the validation cohort

**Model:** Mean intra-SWJ interval ~ Centralized age + Sex + Diagnosis

**Reference group:** Mean age of 64.62 | Male | Control.

| **Variables** | **B** | **SE** | **t-value** | **p** |
| --- | --- | --- | --- | --- |
| Intercept | 160.29 | 39.99 | 4.01 | <0.001 |
| Centralized age | 4.31 | 1.73 | 2.49 | 0.02 |
| Female | 30.00 | 28.73 | 1.04 | 0.30 |
| *PD* | 70.51 | 38.04 | 1.85 | 0.07 |
| *PSP* | 51.035 | 39.55 | 1.29 | 0.20 |

*Note:* B: unstandardized coefficients; SE: standard error

**** Comments:** Mean intra-SWJ interval was not significantly different across groups.

## **Table S4E.** The linear model comparing mean vertical component across diagnoses including age and sex as covariates in the validation cohort

**Model:** Mean vertical component ~ Centralized age + Sex + Diagnosis

**Reference group:** Mean age of 64.62 | Male | Control

| **Variables** | **B** | **SE** | **t-value** | **p** |
| --- | --- | --- | --- | --- |
| Intercept | 0.24 | 0.17 | 1.44 | 0.16 |
| Centralized age | -0.007 | 0.007 | -0.95 | 0.35 |
| Female | 0.17 | 0.10 | 1.67 | 0.10 |
| *PD* | -0.02 | 0.18 | -0.13 | 0.90 |
| *PSP* | -0.09 | 0.16 | -0.56 | 0.58 |

*Note:* B: unstandardized coefficients; SE: standard error

*This model showed the heteroskedasticity, so the cluster robust type-3 was used to estimate the standard error and p-value.

**** Comments:** Mean vertical component was not significantly different across groups.

## **Table S5A.** The linear model exploring the effect of visual acuity on SWJ rate across diagnoses in the discovery cohort

**Model:** SWJ rate ~ LogMAR + Diagnosis

**Reference group:** Control

| **Variables** | **B** | **SE** | **t-value** | **p** |
| --- | --- | --- | --- | --- |
| Intercept | 3.58 | 1.56 | 2.45 | <0.02 |
| LogMAR | 0.35 | 3.99 | 0.09 | 0.93 |
| *AD* | 0.81 | 2.30 | 0.36 | 0.72 |
| *PD* | 3.47 | 2.28 | 1.52 | 0.13 |
| *PSP* | 15.60 | 2.78 | 5.61 | <0.0001 |

*Note:* B: unstandardized coefficients; SE: standard error

**** Interpretation:** LogMAR = log10(Snellen denominator/Snellen numerator). The visual acuity did not associate with the number of SWJ rate.

** This linear regression model was built based on n=47 control, n=28 AD, n=32 PD and n=16 PSP.

## **Table S5B.** The linear model exploring the effect of Levodopa dosage on SWJ rate in Parkinson’s disease group in the discovery cohort

**Model:** SWJ rate ~ LEDD × H-Y

| **Variables** | **B** | **SE** | **t-value** | **p** |
| --- | --- | --- | --- | --- |
| Intercept | 2.80 | 5.61 | 0.50 | 0.62 |
| LEDD | 0.004 | 0.01 | 0.39 | 0.70 |
| H-Y | 2.59 | 3.04 | 0.85 | 0.40 |
| Interaction between LEDD: H-Y | -0.003 | 0.006 | -0.46 | 0.65 |

*Note:* B: unstandardized coefficients; SE: standard error

**** Interpretation:** LEDD: Levodopa equivalent daily dose was calculated using website: <https://www.parkinsonsmeasurement.org/toolBox/levodopaEquivalentDose.htm>

## **Table S5C.** Linear model exploring the effect of ACE-III, Hoen-Yahr stage, and disease duration on SWJ rate in PSP in the discovery cohort

**Model:** SWJ rate ~ Disease duration + ACE-III score + H-Y stage

| **Variables** | **B** | **SE** | **t-value** | **p** |
| --- | --- | --- | --- | --- |
| Intercept | 9.30 | 22.81 | 0.41 | 0.69 |
| ACE-III score | 0.22 | 0.28 | 0.78 | 0.44 |
| Hoehn-Yahr score | -4.57 | 2.60 | -1.76 | 0.09 |
| Disease duration | 0.11 | 1.58 | 0.07 | 0.95 |

*Note:* B: unstandardized coefficients; SE: standard error

**** Interpretation:** There was a negative but not significant relationship between SWJ rate and H-Y in PSP.

**Table S5D.** Exploratory associations between clinical measures and SWJ rate in the validation PSP cohort.

**Model:** SWJ rate ~ each **Clinical measure**

| **Clinical measure** | **Analysed, n** | **Missing,**  **n** | **B (events/min)** | **SE** | **Unadjusted**  **p-value** | **Holm-adjusted p** |
| --- | --- | --- | --- | --- | --- | --- |
| PSPRS | 20 | 5 | 0.10 | 0.40 | 0.80 | 1.00 |
| H–Y stage | 23 | 2 | 5.98 | 5.07 | 0.25 | 1.00 |
| MoCA raw score | 18 | 7 | −0.89 | 1.12 | 0.44 | 1.00 |
| MDS-UPDRS part III | 20 | 5 | 0.31 | 0.31 | 0.32 | 1.00 |
| Disease duration | 24 | 1 | 3.00 | 2.83 | 0.30 | 1.00 |

Each row represents a separate univariable linear regression using participants with available SWJ rate and clinical-measure data. B denotes the unstandardised change in SWJ rate (events/min) per one-point increase in the clinical score or one-stage increase in H-Y stage. Models were not adjusted for other covariates. Standard errors and p-values shown are conventional estimates.

**Table S5E.** Test-retest reliability in the 18 controls with repeat from 3 months to one year in the discovery cohort

| **Parameters** | **SWJ rate per min** | **SWJ amplitude per min** |
| --- | --- | --- |
| Intraclass Correlation Coefficient  (p-value) | 0.947  (p<0.0001) | 0.932  (p<0.0001) |
| Wilcoxon test (p-value) | p = 0.55 | p = 0.55 |

**** Interpretation:** The ICC showed the excellent reliability/agreement in both metrics SWJ rate per min and SWJ amplitude.

## **Table S5F.** Exploratory comparison of SWJ rate between PSP-RS and pooled non-RS phenotypes within each cohort.

| **SWJ rate per min**  **(Mean ± SD)** | **PSP-RS** | **PSP-nonRS** | **p-value two-sided Wilcoxon rank-sum test** |
| --- | --- | --- | --- |
| Discovery cohort | n = 11 | n = 14:  PSP-CBS 5, PSP-F 4, PSP-SL 3, PSP-PGF 1, PSP-P 1 |  |
|  | 20.8 ± 19.96 | 15.21 ± 15.81 | 0.77 |
| Validation cohort | n = 15 | n = 10:  PSP-P 6, PSP-PGF 4 |  |
|  | 28.52 ± 22.73 | 8.36 ± 18.72 | 0.006 |

*Comments: SWJ rates did not differ significantly between these groups in the discovery cohort, but were higher in PSP-RS in the validation cohort.

These exploratory findings suggest that phenotype composition may influence the pooled results, particularly in the validation cohort. However, the small subgroups and differing composition of the non-RS groups preclude firm conclusions about phenotype-specific diagnostic performance. The non-significant discovery comparison should not be interpreted as evidence of equivalence.

**Figure S2.** SWJ rate per min distribution for each PSP phenotype.

## **Table S6.** Calibration results of the nested CV model in the discovery cohort

| **Calibration parameters** | **PSP vs Control** | **PSP vs AD** | **PSP vs PD** | **PSP vs others** |
| --- | --- | --- | --- | --- |
| Brier score | 0.13  (0.09–0.18) | 0.23  (0.18–0.27) | 0.20  (0.16–0.25) | 0.10  (0.07–0.13) |
| Scaled Brier score  (Brier Skill Score) | 0.22  (0–0.40) | 0.07  (-0.15–0.23) | 0.07  (-0.10–0.23) | 0.10  (-0.06–0.25) |
| Calibration intercept | -0.001  (-0.66–0.48) | -0.01  (-0.65–0.55) | 0.01  (-0.58–0.52) | -0.02  (-0.56–0.41) |
| Calibration slope | 0.81  (0.43–2.02) | 0.59  (0.03–2.11) | 0.71  (0.16–1.52) | 0.89  (0.53–1.47) |

## **Figure S3.** Calibration plot across pairs of comparison

Calibration performance is summarised in **Table S6** and **Figure S3**. Overall, the models showed no evidence of systematic over- or underestimation, with calibration intercepts close to zero across all diagnostic comparisons. Calibration slopes were below 1 for all models, suggesting some degree of overfitting or over-dispersion of predicted probabilities, although confidence intervals were wide and generally included 1.

Calibration appeared best for PSP versus controls and PSP versus all other groups, which also showed the lowest Brier scores.

|  |  |
| --- | --- |
| **SWJ rate threshold** \| **Sensitivity** \| **Specificity**; **>10** \| 0.56 \| 0.78; **>24** \| 0.20 \| 0.97 | **SWJ rate threshold** \| **Sensitivity** \| **Specificity**; **>10** \| 0.60 \| 0.54; **>24** \| 0.28 \| 0.90 |

## **Figure S4.** ROC curves using selected definition to differentiate PSP vs PD from the discovery cohort (on the left panel) and the validation cohort (on the right panel)

## **Table S7.** Participant-level diagnostic performance of SWJ in differentiating PSP from other groups using total SWJ amplitude per min in the discovery cohort

The logistic regression model at step 4: **logit[P(PSP)] = β0 + β1×total SWJ amplitude per min**

| **Comparison** | **AUC (95% CI)** | **Se** | **Sp** | **LR+** | **LR−** |
| --- | --- | --- | --- | --- | --- |
| **PSP (n=25) vs control (n=88)** | 0.79  (0.67–0.88) | 0.80  (0.60–0.96) | 0.75  (0.49–0.90) | 3.20  (1.80–7.39) | 0.26  (0.05–0.50) |
| **PSP(n=25) vs PD (n=32)** | 0.62  (0.46–0.76) | 0.56  (0.24–0.92) | 0.81  (0.41–1.00) | 2.62  (1.33–12.8) | 0.56  (0.18–0.84) |
| **PSP (n=25) vs AD (n=35)** | 0.63  (0.49–0.76) | 0.80  (0.36–1.00) | 0.54  (0.23–0.91) | 1.70  (1.21–1.70) | 0.35  (0.00–0.74) |
| **PSP vs others (n=180)** | 0.71  (0.59–0.81) | 0.76  (0.48–0.96) | 0.66  (0.41–0.88) | 2.24  (1.51–4.72) | 0.35  (0.08–0.65) |

**** Comment:** Total SWJ amplitude was not outperformed the SWJ rate in improving diagnostic utility.
